# The effect of role play-based education on nursing students’ stigmatizing attitudes, empathy levels, and communication skills toward the care of individuals with substance use disorder

**DOI:** 10.3389/fpubh.2026.1941630

**Published:** 2026-10-19

**Authors:** Neşe Ataman Bor, Soner Berşe, Sümeyye Akçoban, Ezel Avcı

**Affiliations:** 1 Department of Nursing, Faculty of Health Sciences, Hakkari University, Hakkari, Türkiye; 2 Department of Nursing, Faculty of Health Sciences, Gaziantep University, Gaziantep, Türkiye; 3 Department of Health Care Services, Kırıkhan Vocational School, Hatay Mustafa Kemal University, Hatay, Türkiye; 4 Logos Psychology Clinic, Gaziantep, Türkiye

**Keywords:** communication skills, empathy, nursing education, role-play simulation, stigma, substance use disorder

## Abstract

**Background:**

Stigmatizing attitudes toward individuals with substance use disorder (SUD) remain common among healthcare professionals and students and may negatively affect care quality, therapeutic relationships, and treatment access. Nursing students may demonstrate stigma, limited empathy, and insufficient communication skills in SUD care contexts. Active learning approaches such as role play have been suggested to improve affective and communication-related competencies; however, experimental studies simultaneously targeting stigma, empathy, and communication skills in SUD contexts remain limited.

**Purpose:**

This study aimed to examine the effect of role play–based education on nursing students’ stigmatizing attitudes toward individuals with SUD, empathy levels, and communication skills.

**Materials and methods:**

A quasi-experimental pretest–posttest control group design was conducted during the 2025–2026 academic year with 76 nursing students (experimental *n* = 38; control *n* = 38) at a faculty of health sciences in Türkiye. The experimental group received a structured four-session role play–based SUD education program, while the control group continued with the standard curriculum. Data were collected using the Toronto Empathy Questionnaire (TEQ), Communication Skills Inventory (CSI), and Attitudes Toward Mental Illness Scale—Substance Related Subscale (ATMI-SRS). Linear mixed models were used as the primary analysis, supported by ANCOVA and mixed ANOVA. The Bonferroni-adjusted significance level was set at *α* = 0.017.

**Results:**

Significant time × group interactions were found for all outcomes. The experimental group showed greater reductions in stigmatizing attitudes than controls (*β* = −5.61, *p* < 0.001), with a 57% reduction versus 15% in controls. Empathy increased significantly (*β* = 11.92, *p* < 0.001), and communication skills improved significantly (*β* = 20.06, *p* < 0.001). Effects were stronger among students with higher baseline stigma levels.

**Conclusion:**

Role play–based education appears effective in reducing stigma and improving empathy and communication skills toward individuals with SUD among nursing students. Despite quasi-experimental design limitations, findings support integrating structured role play into nursing curricula. Further randomized controlled studies with larger samples and long-term follow-up are recommended.

## Introduction

Drug use disorders constitute a substantial and evolving global public health burden, with important consequences for individuals, families, and societies (1, 2). The literature indicates that stigmatizing attitudes toward individuals with substance use disorder (SUD) may be prevalent among healthcare professionals and students, negatively affecting quality of care and access to treatment (3, 4). Although nurses play a critical role in addiction care, nursing students have been reported to exhibit negative attitudes, stigmatizing perceptions, and limited communication skills toward individuals with SUD (5, 6). In clinical settings, nurses may perceive patients with a history of substance use as having low motivation and may experience reduced professional role support (7). Additionally, stereotypical beliefs shaped by societal and media influences may sustain stigmatizing attitudes among students when not critically addressed during training (8).

Evidence from the broader mental health literature further supports examining stigma and empathy as interrelated educational and clinical constructs. A study of university students found a significant inverse relationship between negative beliefs toward mental illness and empathy and also reported less negative beliefs among medical students who had completed psychiatry training. More recent research among medical students showed that psychiatry training was associated with higher empathy, while perceived stress was negatively associated with empathy. In addition, research involving dental students has emphasized empathy as a core component of the clinician–patient relationship and highlighted the importance of incorporating strategies to strengthen empathy into health-professions curricula. Taken together, these findings suggest that stigmatizing attitudes and empathy should not be considered in isolation and support educational approaches that address both constructs within health-professions education (9–11).

Among healthcare students, negative attitudes toward stigmatized conditions such as mental illness and addiction may persist despite increasing educational level, potentially affecting the quality of clinical care (5, 12). Community-informed simulation and structured debriefing have been shown to help nursing students recognize stigma, challenge pre-existing biases, foster empathy, and develop more person-centered approaches toward individuals with opioid use disorder (13). Educational research also demonstrates that structured and practice-based learning approaches significantly improve professional competencies such as clinical decision-making, communication, and team role awareness in nursing students (14, 15).

Educational interventions, particularly active learning methods such as simulation and role play, have emerged as effective pedagogical approaches for improving nursing students’ clinical decision-making, communication, empathy, and self-awareness skills (14, 16). The literature reports that role play–based learning increases students’ self-confidence, strengthens communication and clinical skills, promotes active participation in learning, and significantly improves empathy levels (17, 18). A comparative study of role play and simulated patient approaches demonstrated that these methods improve communication behaviors and the quality of patient interaction in different ways, offering complementary advantages for communication training (19).

In the context of addiction and SUD, role play–based interventions have shown promising results in reducing stigmatizing tendencies, strengthening empathetic and patient-centered care approaches, and increasing awareness of personal biases (20, 21). Previous studies indicate that role play and simulation practices produce positive changes in attitudes, empathy, and communication outcomes, while also supporting patient-centered care approaches (13, 22–26). However, healthcare professionals may experience communication difficulties when working with individuals with SUD, and current training programs often remain theoretical, with persistent gaps in applied communication skill development (27–29). Moreover, a systematic review highlights the importance of stigma-reduction training but indicates that randomized and long-term comparative studies specifically focused on role play remain limited (30). Therefore, there is a need for experimental role play–based studies that simultaneously target stigmatizing attitudes, empathy, and therapeutic communication skills (31, 32). Drawing on both the broader mental health literature and SUD-specific evidence, simultaneously addressing stigma and empathy may provide a more comprehensive educational approach to preparing nursing students for person-centered care of individuals with SUD. Accordingly, the present study aims to examine the effects of role play–based education on stigmatizing attitudes, empathy levels, and communication skills toward individuals with SUD among nursing students.

Accordingly, this study seeks to answer the following research questions:

- Does role play–based education reduce stigmatizing attitudes toward individuals with substance use disorder among nursing students compared with standard curriculum education?
- Does role play–based education improve empathy levels among nursing students toward individuals with substance use disorder?
- Does role play–based education improve therapeutic communication skills of nursing students in the care of individuals with substance use disorder?

## Materials and methods

### Study design

This study employed a quasi-experimental pretest–posttest control group design to examine the effect of role play-based education on nursing students’ stigmatizing attitudes toward individuals with substance use disorder (SUD), empathy levels, and communication skills. The experimental group received a structured role play-based education program on SUD, while the control group continued with the standard curriculum without any additional intervention. The STROBE checklist was used to report the study (33).

### Setting and participants

The study was conducted at Hakkari University, Faculty of Health Sciences, Department of Nursing, during the 2025–2026 academic year. The experimental group comprised 38 third-year nursing students, and the control group comprised 38 fourth-year nursing students (total *N* = 76). Sample size was determined using G*Power 3.1 (34) for a two-group two-measurement design with effect size *f* = 0.25, *α* = 0.05, and power = 0.80, yielding a minimum required sample of 72 (36 per group). The target was 40 students per group, with 38 completing the study in each group (Figure 1).

**Figure 1:**
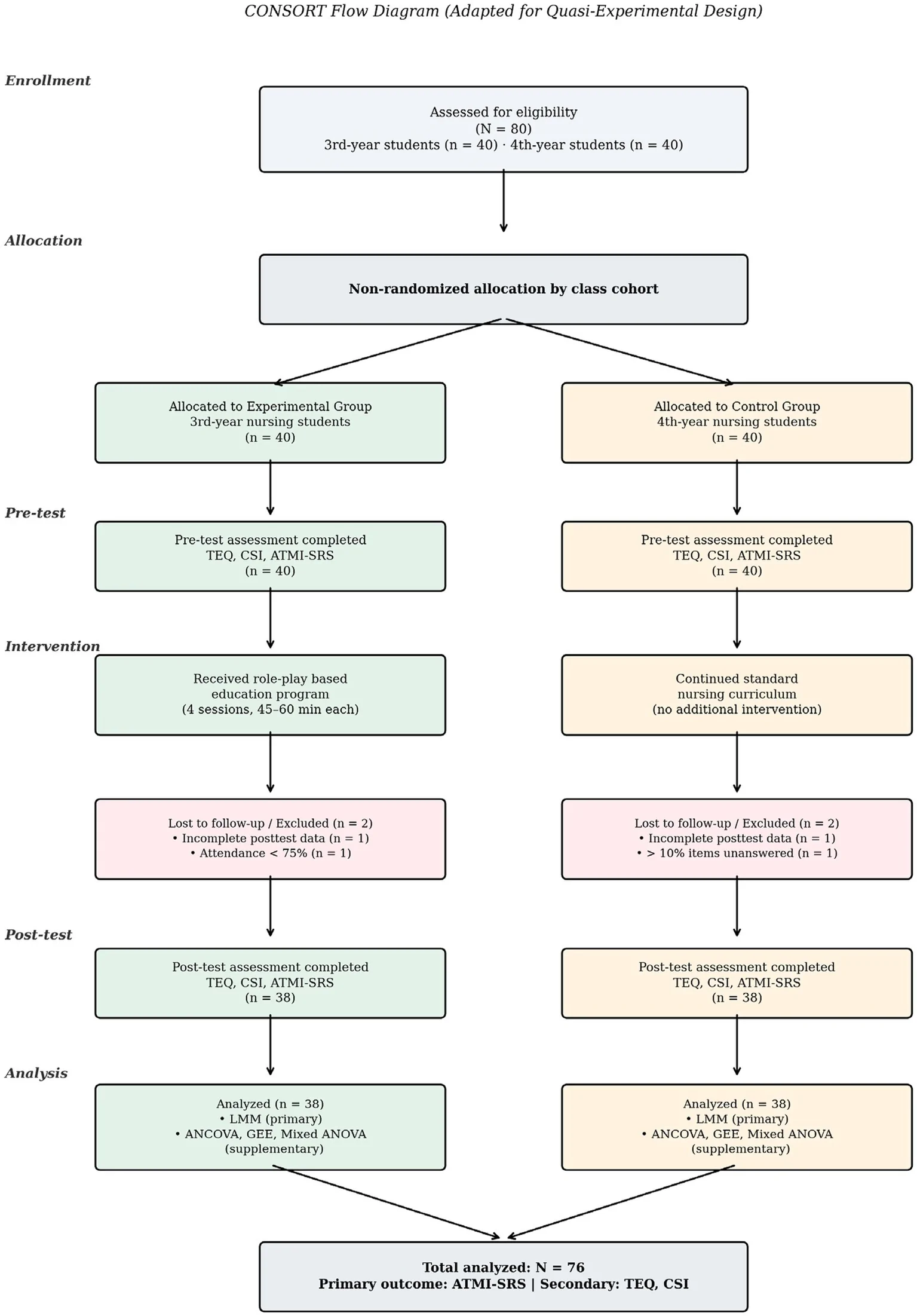
This figure shows the distribution of the experimental and control groups in the study.

#### Eligibility criteria

Students were eligible to participate if they (a) were enrolled in the third or fourth year of the nursing program, (b) were aged 18 years or older, and (c) provided written informed consent. Participants were excluded from the analytic sample if they had missing pretest or posttest data or left more than 10% of questionnaire items unanswered. In the experimental group, attendance at less than 75% of the intervention sessions constituted an additional exclusion criterion.

#### Attrition and exclusion after enrollment

A total of 80 students were enrolled at baseline, with 40 students allocated to the experimental cohort and 40 to the control cohort. Four students were excluded after enrollment. In the experimental group, one student had incomplete posttest data and one attended fewer than 75% of the intervention sessions. In the control group, one student had incomplete posttest data and one left more than 10% of questionnaire items unanswered. Consequently, 38 students in each group (*N* = 76) were included in the final analyses (Figure 1).

#### Missing data

Participants who met the prespecified exclusion criteria for missing pretest or posttest data or excessive missing questionnaire responses were not included in the analytic sample. No outcome-level imputation was performed; the analyses were conducted using the available pretest and posttest data from the 76 participants who remained eligible for analysis after application of these criteria.

## Instruments

### Sociodemographic data form

A researcher-developed sociodemographic data form was used to collect information on age, gender, class level, residence, smoking status, alcohol use, prior SUD theory course, clinical encounters with patients with SUD, perceived competency in SUD care, and family history of SUD. The items were developed by the research team based on the objectives and variables of the present study and were not directly derived from a previously published instrument.

### Toronto empathy questionnaire

The TEQ, developed by Spreng et al. (35) and adapted to Turkish by Totan et al. (36), consists of 13 items rated on a 5-point Likert scale (1 = not at all appropriate to 5 = completely appropriate). Items 1, 3, 5, 7, 8, 9, 11, and 12 are reverse-scored. The original Turkish version reported Cronbach’s *α* = 0.79. In the present study, *α* = 0.84 and *ω* = 0.84 (see Table 1).

**Table 1:** Reliability of study instruments.

Scale	Items	*α*	*ω*
TEQ total	13	0.84	0.84
CSI total	45	0.71	0.83
ATMI-SRS total	21	0.94	0.94

*α* = Cronbach’s alpha; *ω* = McDonald’s omega. Coefficients computed from pretest data (*n* = 76). Subscale reliability coefficients are not reported due to inadequate internal consistency in the present sample; therefore, only total scores were used in inferential analyses. TEQ, Toronto Empathy Questionnaire; CSI, Communication Skills Inventory; ATMI-SRS, Attitudes Toward Mental Illness Scale—Substance Related Sub-scale.

### Communication skills inventory

The CSI, developed by Balcı and Ersanlı (37), comprises 45 items rated on a 5-point Likert scale (1 = never to 5 = always), measuring cognitive, emotional, and behavioral dimensions of communication. The original version reported Cronbach’s *α* = 0.72. In the present study, the total CSI *α* = 0.71 and *ω* = 0.83 (see Table 1). Subscale reliability coefficients were not reported due to inadequate internal consistency in the present sample; therefore, only the total CSI score was retained for inferential analyses.

### Attitudes toward mental illness scale—substance related sub-scale

The ATMI-SRS, developed by Ersoy et al. (38), consists of 21 items (10 related to alcohol, 11 related to other substances), with dichotomous scoring (Agree = 1, Disagree = 0). Total score ranges from 0 to 21, with higher scores indicating greater stigmatizing attitudes toward individuals with SUD. The original version reported Cronbach’s *α* = 0.87. In the present study, *α* = 0.94 and *ω* = 0.94 (see Table 1). Given the binary item format, both parametric and nonparametric approaches as well as binomial generalized estimating equations (GEE) were employed.

### Intervention

The intervention consisted of a 4-session role play-based education program, each session lasting 45 to 60 min, conducted in the nursing skills laboratory. The program utilized three rotating roles: patient, nurse, and observer. Each session followed a structured format comprising a preparation phase, role-play execution, and debriefing. During the preparation phase, the instructor introduced foundational concepts related to addiction, presented realistic clinical scenarios, and assigned participants to roles. Students in the patient role portrayed individuals with various SUD histories and presentations. Students in the nurse role practiced active listening, reflective communication, and motivational interviewing techniques. Observers provided structured feedback based on predefined criteria. The sessions incorporated simulated materials and comprehensive debriefing to consolidate learning.

### Data collection procedure

Both the experimental and control groups completed baseline assessments before the intervention, including the sociodemographic data form, TEQ, CSI, and ATMI-SRS. Following baseline assessment, the experimental group received the 4-session role play-based education program, while the control group continued with the standard nursing curriculum without any additional intervention. Upon completion of the intervention period, both groups completed posttest assessments using the same instruments (TEQ, CSI, ATMI-SRS).

## Data analysis

All analyses were conducted in R (version 2025.05.0 + 496). The primary analytical approach employed linear mixed models (LMM) estimated with restricted maximum likelihood (REML). Models included fixed effects for time (pre/post), group (experimental/control), and the time × group interaction, with a random intercept for participant. The time × group interaction was the parameter of primary interest. Denominator degrees of freedom were estimated using the Satterthwaite approximation, and results are reported as unstandardized coefficients (β) with 95% confidence intervals.

A sensitivity analysis additionally adjusted for age, smoking status, and prior SUD theory course. Because group assignment was based on intact cohorts (third- vs. fourth-year students), age and group were highly collinear (VIF = 27.3). Therefore, unadjusted LMM results are presented as the primary findings, and covariate-adjusted models are reported as supplementary analyses and interpreted cautiously.

As supplementary analyses, analysis of covariance (ANCOVA) was performed with posttest score as the dependent variable, group as the fixed factor, and pretest score as a covariate. Slope homogeneity was tested using a group × pretest interaction. For ATMI-SRS, where this assumption was violated (*p* = 0.009), an interaction ANCOVA model was fitted and conditional effects at selected baseline percentiles were estimated.

Given the dichotomous item structure of ATMI-SRS, a robustness analysis was conducted using binomial generalized estimating equations (GEE) with a logit link and an exchangeable working correlation structure, modeling the number of endorsed items out of 21 (successes = total score; trials = 21). ATMI-SRS was prespecified as the primary outcome, and TEQ and CSI total score as secondary outcomes. A Bonferroni-adjusted significance threshold of α = 0.017 was applied. Effect sizes were computed as Cohen’s d for LMM and partial eta-squared (ηp^2^) for ANCOVA. Distributional assumptions were assessed using the Shapiro–Wilk and Levene’s tests.

### Ethical considerations

The study received approval from the Hakkari University Scientific Research and Publication Ethics Committee (Decision No: 2025/249, Date: December 3, 2025). All participants provided written informed consent prior to enrollment. Participation was voluntary, and enrollment in the study had no impact on academic standing or grades. The study was conducted in accordance with the ethical principles outlined in the Declaration of Helsinki.

## Results

Baseline comparisons revealed significant differences between groups on age (experimental: *M* = 21.84, SD = 1.88; control: *M* = 22.82, SD = 1.23; U = 366.50, *p* < 0.001; Table 2). The experimental group reported lower baseline empathy than the control group. Prior SUD theory course enrollment differed between groups, with 6 students (15.8%) in the experimental group having taken this course compared to none in the control group (Fisher’s exact, *p* = 0.025). Smoking status also differed significantly (*χ*^2^ = 9.80, *p* = 0.007). Other demographic variables did not differ significantly between groups.

**Table 2:** Sociodemographic characteristics of the experimental and control groups.

Variable	Experimental (*n* = 38)	Control (*n* = 38)	Test statistic	*p*
Age (M ± SD)	21.84 ± 1.88	22.82 ± 1.23	U = 366.50	<0.001***
Gender				
Female	24 (63.2)	21 (55.3)	Fisher’s	0.641
Male	14 (36.8)	17 (44.7)		
Residence				
Dormitory	21 (55.3)	15 (39.5)	Fisher’s	0.251
Student house	17 (44.7)	23 (60.5)		
Prior SUD theory course				
Yes	6 (15.8)	0 (0.0)	Fisher’s	0.025*
No	32 (84.2)	38 (100.0)		
Smoking status			Fisher’s	0.003**
Yes	29 (76.3)	34 (89.5)		
No	9 (23.7)	4 (10.5)		
Alcohol use				
Yes	7 (18.4)	11 (28.9)	Fisher’s	0.419
No	31 (81.6)	27 (71.1)		
Clinical SUD encounter				
Yes	6 (15.8)	5 (13.2)	Fisher’s	1.000
No	32 (84.2)	33 (86.8)		
Perceived SUD care competency			*χ*^2^ = 0.63	0.889
Not at all competent	8 (21.1)	6 (15.8)		
Inadequate	16 (42.1)	19 (50.0)		
Undecided	8 (21.1)	8 (21.1)		
Competent	6 (15.8)	5 (13.2)		
Family SUD history				
Yes	6 (15.8)	8 (21.1)	Fisher’s	0.768
No	32 (84.2)	30 (78.9)		

Categorical data are presented as *n* (%). Age is presented as M ± SD for descriptive purposes; the Mann–Whitney U test was used due to non-normal distribution (experimental: Mdn = 21.0, IQR = 21.0–22.0; control: Mdn = 23.0, IQR = 22.0–23.0). For smoking status, expected cell counts were < 5; Fisher’s exact test confirmed significance (*p* = 0.003). *p < 0.05; **p < 0.01; ***p < 0.001. SUD = Substance Use Disorder.

The overall scale reliability ranged from acceptable to excellent. TEQ demonstrated good internal consistency (*α* = 0.84, ω = 0.84), CSI showed acceptable-to-good reliability (*α* = 0.71, *ω* = 0.83), and ATMI-SRS exhibited excellent internal consistency (*α* = 0.94, *ω* = 0.94). Since subscale reliability was insufficient, conducting analyses based on total scale scores was methodologically appropriate (Table 1).

Baseline data indicated equivalent communication skills between groups on the CSI total score (experimental: *M* = 114.79, SD = 11.90; control: *M* = 114.27, SD = 12.77; *t* = 0.18, *p* = 0.856). However, the experimental and control groups showed substantial and statistically significant differences on the TEQ (experimental: *M* = 32.84, SD = 5.36; control: *M* = 49.00, SD = 8.92; Welch’s *t* (60.6) = −9.57, *p* < 0.001), with the experimental group demonstrating considerably lower baseline empathy. Welch’s correction was applied due to unequal variances between groups (Levene’s *F* = 9.89, *p* = 0.002). Stigmatizing attitudes (ATMI-SRS) were equivalent at baseline (U = 740.50, *p* = 0.851; Table 3).

**Table 3:** Pretest and posttest scores by group.

Scale	Experimental pre-test M ± SD	Experimental post-test M ± SD	Control pre-test M ± SD	Control post-test M ± SD	Test	*p*
TEQ Total	32.84 ± 5.36	50.45 ± 7.10	49.00 ± 8.92	54.68 ± 7.85	t(60.6) = −9.57	<0.001***
CSI Total	114.79 ± 11.90	140.39 ± 13.20	114.27 ± 12.77	119.89 ± 12.60	t = 0.18	0.856
ATMI-SRS Total	13.34 ± 6.18	5.74 ± 4.90	13.21 ± 7.01	11.21 ± 6.30	U = 740.50	0.851

TEQ, Toronto Empathy Questionnaire; CSI, Communication Skills Inventory; ATMI-SRS, Attitudes Toward Mental Illness Scale—Substance Related Sub-scale. TEQ t-test employed Welch’s correction (df = 60.6) due to unequal variances (Levene’s F = 9.89, *p* = 0.002). ATMI-SRS: Mann–Whitney U test was used due to non-normal distributions. *p < 0.05; **p < 0.01; ***p < 0.001.

### Distributional assumptions

Distributional assumption checks were conducted prior to inferential analyses. For the primary outcome ATMI-SRS, raw scores deviated from normality in three of four conditions (Shapiro–Wilk *p* < 0.05), and the paired pretest–posttest differences in the experimental group were also non-normal (W = 0.93, *p* = 0.016). A supplementary Wilcoxon signed-rank test confirmed the within-group experimental finding (*Z* = −4.94, *p* < 0.001). For TEQ, Levene’s test indicated unequal variances between groups at both pretest (*p* = 0.002) and posttest (*p* = 0.003); Welch’s correction was applied to the baseline comparison. TEQ posttest distributions showed mild departures from normality in both groups (experimental: W = 0.94, *p* = 0.048; control: W = 0.92, *p* = 0.012). CSI control group posttest scores were non-normal (W = 0.91, *p* = 0.006); all other CSI distributions met normality assumptions. Given that the linear mixed model is robust to moderate violations of normality with n ≥ 30 per group, as reported by Schielzeth et al. (39), and that nonparametric alternatives yielded converging conclusions, parametric results were retained as the primary analysis.

The linear mixed model analysis revealed significant time × group interactions for all three outcome measures (Table 4). On the primary outcome ATMI-SRS, the experimental group demonstrated a significantly greater reduction in stigmatizing attitudes relative to the control group [*β* = −5.61, SE = 1.12, 95% CI (−7.84, −3.37), *t* (74) = −4.99, *p* < 0.001, d = 1.62]. Model-estimated marginal means (EMMs) showed that the experimental group decreased from 13.34 at pretest to 5.74 at posttest (57.0% reduction), while the control group decreased from 13.21 to 11.21 (15.1% reduction).

**Table 4:** Primary analysis: linear mixed model results.

Outcome	*β*	SE	95% CI	*t*	df	*p*	*d*
ATMI-SRS (primary)	−5.61	1.12	[−7.84, −3.37]	−4.99	74	<0.001	1.62
TEQ (secondary)	11.92	2.22	[7.50, 16.34]	5.37	74	<0.001	1.74
CSI Total (secondary)	20.06	3.92	[12.25, 27.87]	5.12	74	<0.001	1.67

LMM with REML estimation. Random intercept per participant. Denominator degrees of freedom estimated via Satterthwaite approximation (N subjects − 2 = 74 for the time × group interaction). β represents the time × group interaction, i.e., the additional pre-to-post change in the experimental group relative to the control group. 95% CI based on the t-distribution. Bonferroni-adjusted *α* = 0.017 for 3 outcomes. All outcomes significant at adjusted threshold. d = Cohen’s d computed from the interaction coefficient divided by pooled residual SD. ATMI-SRS, Attitudes Toward Mental Illness Scale—Substance Related Sub-scale; TEQ, Toronto Empathy Questionnaire; CSI, Communication Skills Inventory.

On the secondary outcome TEQ, the experimental group showed a significantly greater increase in empathy relative to the control group [*β* = 11.92, SE = 2.22, 95% CI (7.50, 16.34), *t* (74) = 5.37, *p* < 0.001, d = 1.74]. EMMs indicated that the experimental group increased from 32.84 to 50.45, while the control group increased from 49.00 to 54.68. On CSI total score, the experimental group demonstrated greater improvement than the control group [*β* = 20.06, SE = 3.92, 95% CI (12.25, 27.87), *t* (74) = 5.12, *p* < 0.001, d = 1.67], with EMMs showing an increase from 114.79 to 140.39 in the experimental group and from 114.27 to 119.89 in the control group.

A sensitivity analysis including age, smoking status, and prior SUD course as covariates confirmed that all three outcomes remained statistically significant at the Bonferroni-adjusted threshold (all *p* < 0.001). However, age and group assignment showed substantial confounding (VIF = 27.3), necessitating cautious interpretation of covariate-adjusted results.

As a robustness check, a binomial GEE analysis of the ATMI-SRS, modeling the proportion of items endorsed (score/21), yielded a significant time × group interaction [*β* = −1.15, SE = 0.23, z = −4.96, *p* < 0.001; OR = 0.32, 95% CI (0.20, 0.50)], indicating lower odds of endorsing stigma items over time in the experimental group relative to controls and providing convergent evidence for the LMM findings.

For the primary outcome ATMI-SRS, testing of the slope homogeneity assumption via a group × pretest interaction revealed heterogeneous regression slopes [*F* (1,72) = 7.32, *p* = 0.009]. An interaction model yielded an interaction term of *β* = −0.23, 95% CI [−0.44, −0.02], *t* = −2.23, *p* = 0.029. Conditional effects analysis revealed that the model-estimated posttest difference (experimental minus control) increased at higher baseline levels: at the 25th percentile (baseline = 8), the difference was −4.27 points; at the 50th percentile (baseline = 15.5), −6.00 points; and at the 75th percentile (baseline = 19), −6.81 points. These conditional effects demonstrate that the intervention effect on stigma reduction is larger for students with higher baseline stigmatizing attitudes.

Within-group paired t-tests revealed large and statistically significant pre-to-post improvements in the experimental group across all measures. For empathy (TEQ), the experimental group increased from *M* = 32.84 to M = 50.45 (*t* = 13.42, *p* < 0.001, *d* = 2.21). The control group also improved significantly but with a much smaller effect size (*t* = 2.75, *p* = 0.009, d = 0.45). For communication skills (CSI total), the experimental group improved from *M* = 114.79 to *M* = 140.39 (*t* = 9.36, *p* < 0.001, *d* = 1.54), whereas the control group change was not statistically significant (*t* = 1.76, *p* = 0.087, *d* = 0.30). For the primary outcome ATMI-SRS, the experimental group demonstrated a substantial reduction in stigmatizing attitudes from *M* = 13.34 to *M* = 5.74 (*t* = 8.43, *p* < 0.001, *d* = 1.39), whereas the control group showed a more modest reduction from *M* = 13.21 to *M* = 11.21 (*t* = 2.99, *p* = 0.005, *d* = 0.49).

A supplementary two-way mixed ANOVA confirmed the primary findings from the LMM analysis. Significant time × group interactions were observed for TEQ [*F* (1,74) = 23.73, *p* < 0.001, ηp^2^ = 0.243], CSI total [*F* (1,72) = 27.12, *p* < 0.001, ηp^2^ = 0.274], and ATMI-SRS [*F* (1,74) = 24.91, *p* < 0.001, ηp^2^ = 0.252]. Large main effects of group were observed for TEQ [*F* (1,74) = 105.60, *p* < 0.001, ηp^2^ = 0.588] and CSI total [*F* (1,72) = 27.23, *p* < 0.001, ηp^2^ = 0.274], reflecting baseline differences. Time main effects were significant for all outcomes [TEQ: *F* (1,74) = 90.58, *p* < 0.001, ηp^2^ = 0.550; CSI total: *F* (1,72) = 62.34, *p* < 0.001, ηp^2^ = 0.464; ATMI-SRS: *F* (1,74) = 73.16, *p* < 0.001, ηp^2^ = 0.497].

Analysis of covariance with posttest scores as the dependent variable, group as the fixed factor, and pretest scores as the covariate confirmed the findings from the mixed models. Significant group effects (favoring the experimental group) were found for TEQ [*F* (1,73) = 11.12, *p* = 0.001, ηp^2^ = 0.132], CSI total [*F* (1,71) = 55.70, *p* < 0.001, ηp^2^ = 0.440], and ATMI-SRS [*F* (1,73) = 62.48, *p* < 0.001, ηp^2^ = 0.461; Table 5].

**Table 5:** Supplementary analysis: analysis of covariance results.

Dependent variable	*F*	df	*p*	ηp^2^
TEQ Total	11.12	1, 73	0.001**	0.132
CSI Total	55.70	1, 71	<0.001***	0.440
ATMI-SRS Total	62.48	1, 73	<0.001***	0.461

Analysis of covariance with posttest score as the dependent variable, group as fixed factor, and pretest score as covariate. ηp^2^ = partial eta-squared. *p < 0.05; **p < 0.01; ***p < 0.001. Slope homogeneity (Group × Pretest interaction) was tested separately: TEQ *F* (1,72) = 0.66, *p* = 0.420; CSI Total *F* (1,70) = 1.27, *p* = 0.264; ATMI-SRS *F* (1,72) = 7.32, *p* = 0.009. Levene’s test of homogeneity of variance: TEQ *p* = 0.003 (unequal variances); all others *p* > 0.05. TEQ, Toronto Empathy Questionnaire; CSI, Communication Skills Inventory; ATMI-SRS, Attitudes Toward Mental Illness Scale—Substance Related Sub-scale.

Slope homogeneity (Group × Pretest interaction) was tested for all outcomes. Homogeneous regression slopes were confirmed for TEQ [*F* (1,72) = 0.66, *p* = 0.420] and CSI total [*F* (1,70) = 1.27, *p* = 0.264]. However, for ATMI-SRS, the assumption of slope homogeneity was violated [*F* (1,72) = 7.32, *p* = 0.009], indicating that regression slopes differed significantly between groups. This violation was addressed through the conditional effects analysis reported in the primary results.

Levene’s test of homogeneity of variance revealed unequal variances for TEQ (*p* = 0.003). All other outcomes met the assumption of homogeneity of variance.

## Discussion

This study evaluated changes in nursing students’ stigmatizing attitudes toward individuals with substance use disorder (SUD), empathy levels, and communication skills following role play–based education. The experimental group showed greater pre-to-post improvements across all three outcomes compared with the control group. Accordingly, the results obtained from this study were interpreted in light of the existing literature.

At baseline, a significant difference was found between groups in terms of empathy levels. The experimental group demonstrated lower empathy scores compared with the control group, whereas communication skills and stigmatizing attitudes were comparable between groups at baseline. Recent studies suggest that experiential and simulation-based educational approaches may produce more pronounced improvements, particularly among students with initially lower empathy levels (40, 41). In educational research, it is recommended that baseline differences be controlled using advanced statistical methods (42). However, the experimental and control groups represented different academic-year cohorts, with third-year students comprising the experimental group and fourth-year students comprising the control group. Differences in academic exposure, clinical experience, and professional development between these cohorts may have influenced empathy, stigmatizing attitudes, and communication skills independently of the intervention. Because academic year/cohort was inseparable from group assignment, statistical adjustment cannot fully distinguish a cohort effect from an intervention effect. Therefore, the observed between-group differences cannot be attributed solely to the role play–based intervention, even for outcomes that were comparable at baseline.

The experimental group showed a significantly greater reduction in stigmatizing attitudes toward individuals with SUD than the control group. While a marked decrease was observed in the experimental group, only limited change was detected in the control group. The literature indicates that educational approaches involving direct contact and experiential learning reduce stigmatizing attitudes toward SUD among healthcare students (25, 43, 44). In particular, role play and simulation-based training have been reported to be effective in modifying stigmatizing attitudes (45, 46). These findings suggest that participation in role play–based education may help students recognize their biases toward individuals with SUD and support the re-evaluation of these attitudes; however, the observed changes should be interpreted in light of the potential cohort effect. The reduction of stigmatizing attitudes is considered an important outcome for promoting ethical, empathetic, and patient-centered care.

In this study, empathy levels increased significantly in the experimental group. However, despite the greater pre-to-post increase observed in the experimental group, the control group maintained a higher mean TEQ score at posttest (54.68 vs. 50.45). Thus, the observed change reflects a substantial narrowing of the baseline empathy gap rather than a reversal or complete elimination of the between-group difference. Although the magnitude of this change was large, interpretation requires particular caution because of the substantial baseline difference in empathy and the confounding between academic year/cohort and intervention group. Because the control group consisted of fourth-year students, they had completed approximately one additional year of nursing education and may have had greater cumulative clinical exposure and developmental maturity than the third-year students in the experimental group. These cohort-related differences may have contributed to the higher baseline TEQ scores observed in the control group and may also partly explain why the control group retained a higher absolute empathy score at posttest. However, developmental maturity, cumulative nursing coursework, and clinical exposure were not measured separately; therefore, their individual contributions to the observed differences in empathy cannot be determined. The lower baseline empathy score in the experimental group may also have allowed greater scope for improvement and may have contributed to the magnitude of the observed change. Accordingly, the larger increase in empathy should not be interpreted as indicating that the experimental group achieved higher absolute empathy levels than the control group at posttest. Educational approaches based on role play and reflective learning have been shown to be effective in enhancing empathy among students (47, 48). Within this context, directly experiencing the patient role may contribute to empathic understanding at both cognitive and emotional levels, although the present design does not allow this change to be attributed independently to the intervention.

Communication skills also improved significantly and robustly in the experimental group, whereas only limited change was observed in the control group. Cortés-Rodríguez et al. (49) found that role play training significantly increased interprofessional communication competence scores among nursing students. In addition, a systematic review by Maghsoodi et al. (17) reported that role-playing techniques improve nursing students’ communication skills, self-efficacy, and clinical performance. The pattern observed in the present study is consistent with evidence suggesting that role play may support the development of communication skills through practice in a safe and controlled learning environment. Nevertheless, because the two groups belonged to different academic-year cohorts, differences in prior educational and clinical experience may also have contributed to the observed improvement.

Finally, the estimated reduction in stigmatizing attitudes was greater among students with higher baseline stigma levels. Previous reviews have identified educational and contact-based approaches as promising strategies for reducing stigma related to substance use disorders and stigma in healthcare settings (50, 51). However, the moderating role of baseline stigma remains insufficiently established. Therefore, the greater estimated reduction observed among students with higher baseline stigma levels in the present study should be considered hypothesis-generating. The single-center quasi-experimental design does not support using baseline stigma levels to guide targeted educational implementation. Further adequately powered randomized studies are needed to examine whether baseline stigma moderates the response to role play–based education.

## Conclusion

This study found that participation in role play–based education was associated with greater reductions in stigmatizing attitudes toward individuals with substance use disorder and greater improvements in empathy and self-reported communication skills among nursing students compared with the control condition. The findings showed significant improvements with large effect sizes across all three main outcome variables in the experimental group. However, because the experimental and control groups consisted of different academic-year cohorts, the observed changes cannot be attributed exclusively to the intervention. Differences in academic and clinical experience between third- and fourth-year students may also have contributed to these outcomes.

Within these methodological constraints, the findings suggest that structured role play may represent a promising experiential educational approach for addressing stigma, empathy, and self-reported communication skills in SUD-related nursing education. However, clinical performance, quality of care, and patient outcomes were not assessed in the present study; therefore, whether these educational improvements translate into actual clinical practice and patient care remains to be determined. Furthermore, the larger estimated reduction in stigma among students with higher baseline stigma levels should be interpreted as an exploratory finding rather than as evidence supporting targeted educational implementation. Future studies should examine whether baseline stigma moderates students’ responses to role play–based education. Rather than supporting immediate curricular integration, these findings provide a basis for further evaluation of structured role play in more rigorously controlled studies.

For future research, randomized controlled designs including multiple universities and larger sample sizes are recommended. Additionally, longitudinal follow-up studies are needed to evaluate the long-term sustainability of the observed improvements following role play–based education. Future studies may also examine the adaptation of role play interventions to different clinical fields and their integration with simulated patients, digital simulation technologies, and interprofessional education models. Moreover, adequately powered randomized studies should investigate whether baseline stigma and empathy levels moderate responses to role play–based education before these characteristics are considered for tailoring educational interventions. Finally, future studies should determine whether improvements in stigma, empathy, and self-reported communication skills translate into observable clinical practice, improved quality of care, and better patient outcomes.

## Limitations

The present study employed a quasi-experimental design with intact class cohorts assigned to experimental and control conditions. This design introduces significant limitations regarding causal inference. The experimental group comprised third-year students while the control group comprised fourth-year students; therefore, academic year/cohort was completely confounded with intervention group, and age was also strongly associated with group assignment. Because cohort and intervention status were perfectly aligned, the independent contribution of academic year could not be statistically separated from that of the intervention. Differences between third- and fourth-year students in academic exposure, clinical experience, and professional development may have influenced not only empathy but also stigmatizing attitudes and communication skills independently of the intervention. Furthermore, baseline differences were substantial on empathy (TEQ), with the experimental group demonstrating considerably lower baseline empathy than the control group (difference ≈ 16 points, d = 2.20, *p* < 0.001). Although linear mixed models and ANCOVA provide some statistical control via regression adjustment, these methods cannot fully address unmeasured confounders or potential interactions with unobserved variables, as noted by Shadish et al. (52). More importantly, statistical adjustment cannot resolve the complete confounding between academic year/cohort and intervention group in the present design. Additional baseline differences in prior SUD course enrollment and smoking status further complicate causal interpretation.

The magnitude of observed effect sizes (d = 1.62–1.74 for the primary outcomes) substantially exceeds the typical effect sizes reported in nursing education interventions, which generally range from d = 0.30 to d = 0.80. Such large effect sizes raise questions regarding potential sources of inflation. First, the substantial baseline differences on TEQ may introduce regression-to-the-mean effects, wherein the experimental group’s lower baseline scores could partially explain larger gains independent of the intervention. Second, differences in academic year and accumulated clinical experience may have contributed to between-group differences in change across the study outcomes. Third, the study employed no blinding procedures; both participants and assessors knew group assignment, creating potential for expectancy effects and social desirability bias. Fourth, the same individuals who delivered the intervention also assessed outcomes, introducing the risk of experimenter bias. Fifth, there is potential for contamination between groups if students discussed the intervention with control group peers. Replication using randomized controlled designs with blinded assessment is essential before drawing firm causal conclusions.

For the primary outcome ATMI-SRS, the assumption of slope homogeneity was violated (*p* = 0.009), indicating that regression slopes differed between groups. A conditional effects analysis demonstrated that the estimated between-group difference in stigma reduction varied across the baseline distribution, with larger estimated differences for students with higher baseline stigmatizing attitudes. The differential conditional effects at the 25th percentile (Δ = −4.27), 50th percentile (Δ = −6.00), and 75th percentile (Δ = −6.81) suggest that a single mean difference may not adequately represent the between-group contrast across all subgroups. Nonetheless, binomial GEE [OR = 0.32, 95% CI (0.20, 0.50)] and conditional effects analyses provided convergent evidence of between-group differences in change, lending some robustness to the statistical findings. However, these convergent analyses do not eliminate confounding by academic year/cohort and therefore should not be interpreted as establishing an independent causal intervention effect.

The study was conducted at a single university located in southeastern Turkey with a sample of n = 38 per group. Although this sample size met the power requirement for detecting medium-sized effects (*f* = 0.25), it is underpowered for detecting smaller interactions or testing moderated effects. The use of intact class cohorts limits generalizability to other educational settings and populations. Multi-site randomized controlled trials with larger sample sizes and broader demographic representation are necessary to establish the generalizability and robustness of the intervention.

Despite these limitations, the convergence of findings across multiple analytical approaches—linear mixed models, ANCOVA, mixed ANOVA, conditional effects analysis, and binomial GEE—provides preliminary evidence of an association between participation in the role play–based intervention and improvements in stigmatizing attitudes, empathy, and communication skills among nursing students. However, because academic year/cohort could not be separated from intervention group, these findings should not be interpreted as demonstrating an independent causal effect of the intervention. Replication in randomized designs with adequate blinding, larger sample sizes, and extended follow-up periods is essential before considering curricular integration of this intervention in nursing education programs.

## Data Availability

The original contributions presented in the study are included in the article/supplementary material, further inquiries can be directed to the corresponding author/s.
